# Exercise reverses the sedentary cardiac phenotype in obesity: a systematic review and meta-analysis of hemodynamic, structural, and functional adaptations

**DOI:** 10.1186/s13102-026-01822-6

**Published:** 2026-06-24

**Authors:** Ahmet Kurtoğlu, Musa Türkmen, Ertuğrul Kurtoğlu, Bekir Çar, Karuppasamy Govindasamy

**Affiliations:** 1 Department of Coaching Education, Faculty of Sport Science, Bandirma Onyedi Eylül University, Balıkesir, Türkiye; 2 Department of Physical Edication and Sport Teaching, Faculty of Sport Science, Inonu University, Malatya, Türkiye; 3 Department of Cardiology, Medical Faculty, Malatya Turgut Ozal University, Malatya, 44210 Türkiye; 4 Department of Recreation, Faculty of Sport Science, Gazi University, Ankara, 06330 Türkiye; 5 Department of Sports, Recreation and Wellness, Symbiosis International (Deemed University), Hyderabad Campus, Modallaguda (V), Nandigama (M), Rangareddy, Hyderabad, Telangana 509217 India

## Abstract

**Background and aim:**

Sedentary lifestyle and obesity are considered to be significant risk factors that create a pathway for the appearance of the ‘sedentary cardiac phenotype’ consisting of cardiac atrophy, myocardial stiffening, and altered haemodynamics. Although exercise training has the potential to reverse this detrimental process, the literature data on the magnitude of improvements and the certainty of evidence are inconsistent. This systematic review and meta-analysis aimed to evaluate the effects of exercise interventions on cardiac morphology, systolic/diastolic function, and haemodynamics in sedentary and obesity-prone individuals.

**Method:**

In accordance with the PRISMA guidelines, the study was conducted by searching the PubMed, Web of Science, and Scopus databases from 1990 to 2025 without applying any filters, using Covidence software. As a result of this comprehensive search, 12 randomised controlled trials (RCTs; N=477) comparing exercise training with a control group in sedentary individuals were included in the analysis. Data were pooled using the Standardised Mean Difference (SMD) and a random-effects model. Publication bias and methodological robustness of the results were tested using the Egger regression test, the Trim-and-Fill method, and Leave-One-Out sensitivity analysis. The certainty of the evidence was graded using the GRADE system.

**Results:**

Exercise training was associated with significant reductions in resting HR and SBP, indicating robust improvements in the haemodynamic profile. Notably, stroke volume (SV) demonstrated a highly significant increase. While global LVEF improvements remained at the statistical threshold, myocardial contractility—measured by the S' wave—improved significantly. Furthermore, exercise induced a physiological, athlete’s heart-like eccentric hypertrophy, evidenced by significant increases in LVMass and LVEDV without pathological wall thickening. Diastolic adaptations were also marked by substantial improvements in the E/A ratio and IVRT. Subgroup analyses indicated that high-intensity interval training (HIIT) was the superior modality for diastolic and structural adaptations, whereas aerobic exercise was the most effective for blood pressure reduction. Importantly, meta-regression analyses revealed two key findings: first, improvements in blood pressure and diastolic function occurred independently of weight loss; second, structural and functional adaptations were linearly associated with improvements in body composition.

**Conclusion:**

Exercise training may reduce selected cardiovascular adverse effects associated with obesity and a sedentary lifestyle, particularly in terms of haemodynamic and diastolic parameters. Structural cardiac adaptations, however, may be more closely linked to changes in BMI. Findings specific to HIIT and aerobic training modalities are exploratory in nature and should be interpreted with caution due to heterogeneity and the limited number of studies.

**Trial registration:**

The systematic review protocol was preregistered in PROSPERO (CRD420261292046).

**Supplementary Information:**

The online version contains supplementary material available at https://doi.org/10.1186/s13102-026-01822-6.

## Introduction

Failure to meet minimum activity guidelines and a sedentary lifestyle, which means occasional, random participation in physical activities [1] has become more pronounced with the increase in factors that make our lives easier [2]. A sedentary lifestyle can be said to be one of the greatest public health problems of our time [3]. In particular, the weakening of physical health due to inactivity invites an increase in diseases such as obesity and type-2 diabetes, as well as various health problems such as coronary heart disease or hypertension, which result in disability or death [3, 4].

Regular physical activity plays an important role in maintaining a healthy lifestyle and both preventing and improving various illnesses [5–7]. It is known that the risk factors are significantly reduced, particularly due to cardiovascular adaptations resulting from long-term regular exercise [8, 9]. To understand these protective effects of exercise on cardiac morphology and haemodynamics, studies have focused primarily on the left ventricle (LV), with parameters such as left ventricular end-diastolic volume (LVEDV) being considered an important guide in determining low cardiorespiratory fitness [10]. A review of the literature reveals numerous primary studies focusing on the effects of different exercise types on the LV in sedentary individuals. For example, step aerobic exercise has been reported to improve structural parameters such as left ventricular end-diastolic diameter (LVEDD), intraventricular septal thickness (IVS), and left ventricular posterior wall thickness (LVPW), as well as systolic and diastolic function parameters. Furthermore, while core exercise has been reported to improve LVEDD structural parameters along with systolic function [11], structured endurance [12] and resistance exercises have been reported to cause an increase in LVEDV [13].

Additionally, high-intensity interval training (HIIT) has been found to improve the E/A ratio, an indicator of diastolic function, and stroke volume, while moderate-intensity continuous training (MICT) has been found to increase relative wall thickness (RWT) and aerobic power [14]. However, the fact that these studies show significant differences in terms of protocol, duration and participant characteristics (gender, age, obesity) may lead to marked heterogeneity in the results. This situation makes it difficult to reach a clear conclusion regarding the relative effectiveness of different exercise modalities on the left ventricle and causes apparent inconsistencies between the findings. Meta-analysis studies conducted within the scope of this subject focus more on patients [15, 16] and athlete [17, 18] while a few studies have focused solely on endurance exercises [19, 20].

In this context, the primary objective of the current systematic review and meta-analysis is to quantitatively synthesise the chronic effects of different exercise modalities (HIIT, Aerobic, Resistance and Team Sports) on cardiac morphology, diastolic function, and haemodynamic profile, and to reveal the physiological modification capacity of the “sedentary cardiac phenotype”. The primary hypothesis of our study is that cardiac remodelling is not a homogeneous process; structural hypertrophy (LVMass), filling dynamics (E/A) and autonomic balance (HR, Blood Pressure) will exhibit independent dose-response relationships depending on the type, intensity, weekly frequency and total duration of exercise. Furthermore, to test the frequently debated “necessity of weight loss” paradigm in obesity management, a secondary mechanistic hypothesis will be examined: whether cardiovascular adaptations progress independently of or in parallel with BMI changes. Supported by advanced sensitivity analyses and meta-regression models, this study aims not only to resolve the heterogeneity in the literature but also to provide a clinical basis for developing evidence-based and targeted exercise prescriptions that maximise cardiovascular protection in sedentary and obesity-prone populations.

## Method

### Registration of systematic review and meta-analysis protocol

This systematic review and meta-analysis study was conducted in accordance with the Preferred Reporting Items for Systematic Reviews and Meta-Analyses (PRISMA) guidelines [21, 22]. The PRISMA checklist for this study is included as electronic supplementary material in Table S1. The study protocol has been registered in the International Prospective Register of Systematic Reviews (PROSPERO) system under the number CRD420261292046.

### Eligibility criteria

Peer-reviewed journal articles written in English or Turkish that examine the effect of exercise interventions on left ventricular hypertrophy in sedentary individuals who occasionally engage in physical activity in a random manner that does not meet minimum activity guidelines have been considered [1]. Review or descriptive articles, conference proceedings, and studies with samples consisting only of athletes or patients who have had myocardial infarction or high hypertension were excluded. In addition, no restrictions were applied in terms of country, time period, or study design. Studies that included left ventricular hypertrophy among cardiac parameters were included. The inclusion and exclusion criteria for this study are shown in Table 1.

**Table 1:** Inclusion and exclusion criteria according to the PICOS method

	Inclusion criteria	Exclusion Criteria
Population	Sedentary individuals, individuals with mild hypertension, or sedentary obese individuals	Athletes, patients with myocardial infarction, patients with high blood pressure
Intervetion	Exercise interventions including pre-test and post-test assessments, randomised controlled trials, experimental studies	Cross-sectional studies,
Comparison	The pre-test and post-test cardiac hypertrophy parameters were compared.	Cross-sectional studies
Outcomes	Cardiac parameters related to left ventricular hypertrophy. Left ventricular hypertrophy, left ventricular mass, ventricular hypertrophy	Studies lacking data on left ventricular parameters among cardiac parameters
Study Design	Randomised controlled trials, experimental studies (pre-test-post-test intervention studies).	Compilations, review articles, conference proceedings, cross-sectional studies.

### Operational definitions

For the purpose of this meta-analysis, predefined operational criteria were utilized to ensure cohort homogeneity. Sedentary status was defined in accordance with the American College of Sports Medicine (ACSM) guidelines as individuals not engaging in at least 30 min of moderate-intensity physical activity on at least 3 days of the week for the preceding 3 months, or explicitly reported as engaging in less than 150 min of moderate-to-vigorous physical activity per week. Overweight and obesity risk criteria were established using the World Health Organization (WHO) body mass index (BMI) thresholds, defining overweight as a baseline BMI of 25.0 to 29.9 kg/m² and obesity as a BMI of ≥ 30.0 kg/m², or an equivalent high body fat percentage as reported by the primary authors.

### Search strategy and selection of studies

An extensive systematic literature search was carried out using three major databases, namely PubMed (MEDLINE), Web of Science (Core Collection), and Scopus, for the period January 1990 to 2025. The search strategy development followed PRISMA 2020 recommendations in detail. The search strategy included both controlled terms (MeSH terms, etc.) and uncontrolled terms used in titles/abstracts ([tiab] or TS=Topic). The concepts included in the search were four main concepts interconnected through Boolean operators (AND/OR): (1) Population (e.g., “sedentary”, “physical inactivity”), (2) Intervention (e.g., “aerobic training”, “HIIT” ), (3) Outcome variables (e.g., “echocardiography”, “left ventricular mass”), and (4) Study Design (e.g., “cohort”, “prospective”). Full search strings (queries) for each database to meet the requirements of PRISMA 2020 reporting guidelines are presented in Supplementary Material 1.

**Fig. 1:**
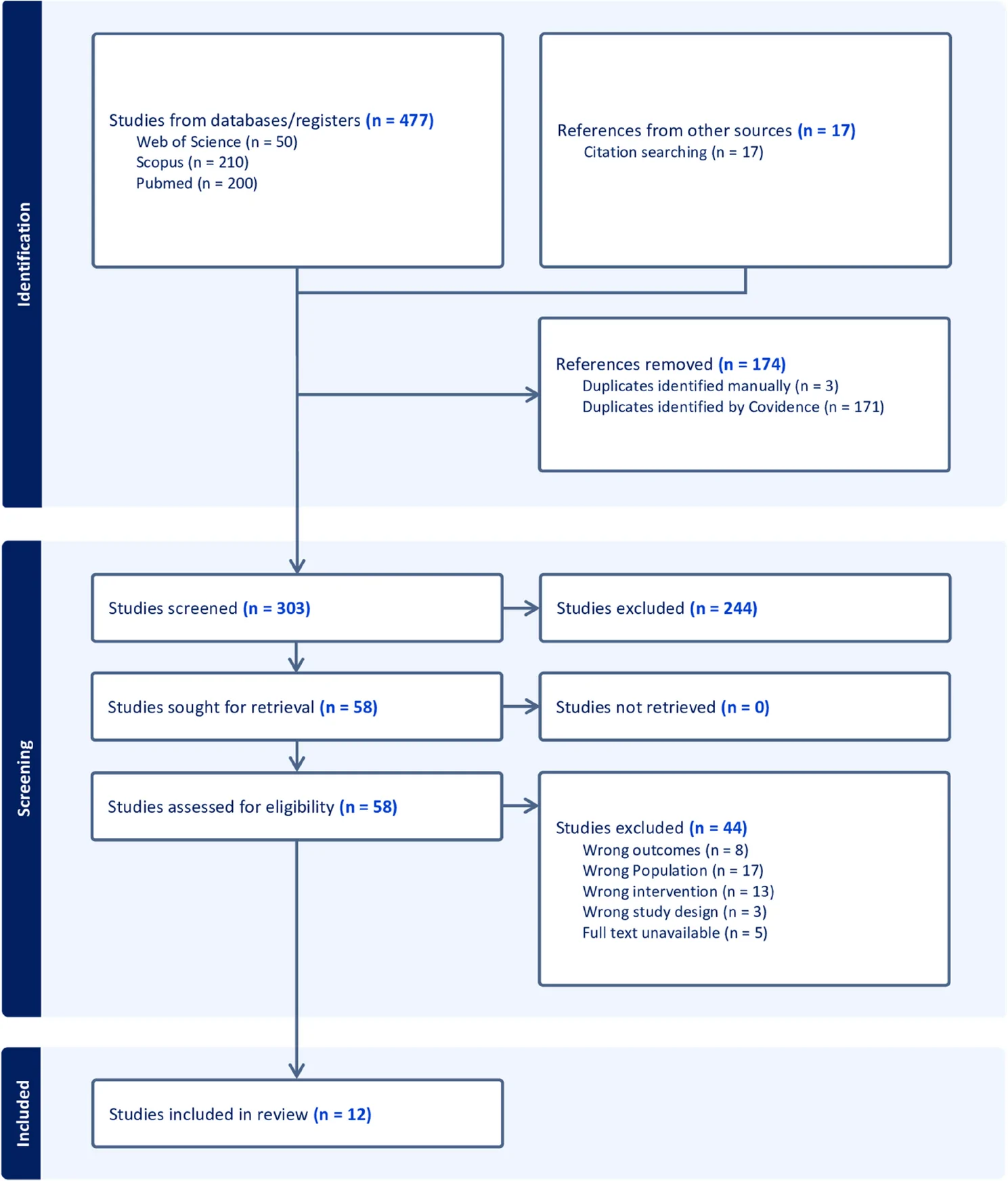
PRISMA flow diagram

After the initial database search, all the citations obtained were exported and imported into Covidence systematic review software (Veritas Health Innovation, Melbourne, Australia) to be automatically checked for duplication and then screened. The primary screening of titles and abstracts was done independently by two reviewers (AK and MT). In case of any disagreement, both reviewers would come to an agreement on their own. Thereafter, the full-text articles that met the eligibility criteria were selected, resulting in a total of 14 articles included in the study. The complete study selection process is illustrated in the PRISMA 2020 flow diagram (Fig. 1).

### Data extraction process

After the final studies were determined, the data extraction phase was initiated. The data extraction form was prepared based on the Data Extraction and Assessment Form in the Cochrane Handbook [23]. After both researchers collected the data, disagreements were resolved through compromise and the data were combined. During the data extraction process of the study, the means and standard deviations related to the dependent variables were transferred to a Microsoft Excel spreadsheet, and the following headings were determined for the other data: Study year, country where the study was conducted, study design, number of participants, participant characteristics, exercise type, exercise intensity, weekly exercise frequency, single session duration, total duration, weekly exercise volume, average age of participants, BMI values, smoking rate among participants, diabetes mellitus rate, SBP, DBP, HR, LVEDV, LVESV, LVEDD, LVESD, EF, IVSd, IVSs, PWT, LVM, LAD, LAV, E, A, S, E’, A’, S’, E/A, E/E’, IVRT, IVCT, and SV average values.

### Quality assessment

The Risk of Bias 2 (RoB 2) tool developed by The Cochrane Collaboration was used to assess the methodological quality and risk of bias of the articles included in the study. Table 2 presents the RoB 2 assessment results for the studies included in the research. This tool evaluates articles in five main areas from a methodological perspective (bias arising from randomization, bias arising from planned interventions, bias arising from incomplete outcome data, bias arising from outcome measurement, bias arising from reporting of outcomes). The results obtained from the evaluation of these areas help us understand whether the results of the studies can be trusted [24].

**Table 2:** The RoB 2 assessment results of the studies

Authors and Date	1	2	3	4	5	Overall
Andersen L.J. et al.,2010 [25]	Low risk	Some concerns	Low risk	Low risk	Low risk	Some concerns
Andersen L.J. et al.,,2014 [26]	Low risk	Some concerns	Low risk	Low risk	Low risk	Some concerns
Baglivo et al., 1990 [27]	High risk	High risk	Low risk	Some concerns	Some concerns	High risk
Howden et al., 2018 [28]	Low risk	Low risk	Low risk	Low risk	Low risk	Low risk
Huang et al., 2019 [29]	Low risk	Low risk	Low risk	Low risk	Low risk	Low risk
Norha et al., 2025 [30]	Low risk	Some concerns	Low risk	Low risk	Low risk	Low risk
Oxborough et al., 2019 [31]	Low risk	Low risk	Low risk	Low risk	Low risk	Low risk
Scharf et al.,2015 [32]	Low risk	Low risk	Some concerns	Low risk	Low risk	Some concerns
Schmidt et al., 2014 [33]	Low risk	Low risk	Low risk	Low risk	Low risk	Low risk
Sjúrðarson et al., 2024 [34]	Low risk	Low risk	Low risk	Low risk	Low risk	Low risk
Spence et al., 2011 [35]	Low risk	Low risk	Low risk	Low risk	Low risk	Low risk
Zheng et al., 2019 [45]	Low risk	Low risk	Some Concerns	Low risk	Low risk	Some concerns

Domain 1: Randomisation process, Domain 2: Alterations from planned interventions, Domain 3: Missing outcome data, Domain 4: Measurement of outcome, Domain 5: Selection of the reported result

### Statistical analyses

All statistical analyses and data visualisations were performed using the R Statistical Software Environment (R Foundation for Statistical Computing, Vienna, Austria). The ‘meta’ [36] and “metafor” [37] packages were used for meta-analysis procedures, while the ‘dmetar’ library was used for publication bias and sensitivity analyses. The analysis process was conducted in full compliance with the guidelines of the Cochrane Handbook for Systematic Reviews of Interventions [23].

To ensure methodological rigor and computational accuracy, only studies reporting complete pre- and post-intervention variance (SD) data were included in the analyses; studies lacking explicit standard deviations were systematically excluded to prevent estimation errors. The mean change values and standard deviations (SD) before and after the intervention were used for continuous variables (e.g., LVEF, LVMass, E/A ratio, BMI). To normalise measurement methods and scale differences between studies, the Standardised Mean Difference (SMD) and 95% Confidence Interval (CI) were calculated as effect size metrics [38]. Specifically, Hedges’ g formulation was applied to the SMD to correct for potential upward bias common in small sample sizes. Furthermore, to provide a more comprehensive assessment of treatment effects across different clinical settings, 95% Prediction Intervals (PI) were computed to represent the expected range within which the true effect size of a future study would fall. When primary studies reported only pre- and post-intervention means and standard deviations (SDs) without the SD of the change score (SD_change), we imputed the missing SD_change values using the standard Cochrane Handbook formula:

$$\:S{D}_{change}=\sqrt{S{D}_{pre}^{2}+S{D}_{post}^{2}-2\cdot\:r\cdot\:S{D}_{pre}\cdot\:S{D}_{post}}$$

For this calculation, a conservative pre-post correlation coefficient (r) of 0.5 was imputed. To verify the robustness of this imputation, an additional sensitivity analysis was conducted by recalculating the pooled effect sizes with alternative correlation coefficients (*r* = 0.3 and *r* = 0.7).

Due to the expected clinical and methodological heterogeneity between exercise protocols and participant populations (sedentary, at risk of obesity, etc.), the Random-Effects Model and Inverse Variance method, which offer a more conservative approach than fixed effects, were preferred in all pooling procedures [39]. The Restricted Maximum Likelihood (REML) estimator was specifically employed to estimate and quantify the between-study variance, which is reported as the τ² (Tau-squared) statistic.

To investigate inter-study statistical heterogeneity, the Cochran Q test (*p* < 0.10) and the I² statistic, which measures the percentage of variation due to factors other than chance, were used. The level of heterogeneity between studies was defined as low, moderate, and high for an I² statistic value between 33%, between 33% and 66%, and above 66%, respectively [40]. To explore possible reasons for heterogeneity (e.g., influence independent of BMI, dose-response relationship), subgroup analysis for categorical variables (Exercise Type: HIIT/Aerobic) and univariate meta-regression analysis for continuous variables (Total Weeks, Frequency, BMI Change) were carried out.

To test the methodological robustness of the findings and determine whether the overall result of a single study was artificially influenced (influential cases), a “Leave-One-Out” sensitivity analysis was applied. In this procedure, one study was removed from the pool at each step, and the overall effect size and statistical significance (p-value) were iteratively recalculated [37].

Publication bias risk was examined using a multidimensional approach. Visual asymmetry was assessed using Funnel Plots; statistical asymmetry was tested using Egger’s linear regression test and Begg’s rank correlation test (*p* < 0.05 was considered significant) [41]. Rosenthal’s Fail-Safe N (FSN) number was calculated to measure the robustness of the results against unpublished “negative” studies [42]. For parameters with asymmetry detected in the funnel plot (e.g., HR, E/A), Duval and Tweedie’s “Trim-and-Fill” method was applied to estimate the adjusted effect size by simulating missing studies [43].

The certainty level of evidence (Certainty of Evidence) obtained for each outcome was assessed using the GRADE approach (Grading of Recommendations Assessment, Development and Evaluation) and rated as High, Moderate, Low, or Very Low based on the risk of error, inconsistency (I^2^), indirectness, uncertainty, and publication bias [44]. In all analyses, the statistical significance level was set at *p* < 0.05 (two-tailed).

## Results

The characteristics of the studies included in the review are presented in Table 3. This meta-analysis included a total of 12 studies conducted between 1990 and 2025 [27] and [30]). The geographical distribution of the studies is quite widespread and includes data from European countries such as Denmark, Finland, Germany, the UK, and the Netherlands; North American countries such as the USA; Asian countries such as China and Taiwan; South American countries such as Argentina; and the African country of South Africa.

**Table 3:** The characteristics of the included studies

Study ID	Year	Country	Exercise Type	*n*	Age (Years)	Male (%)	BMI (kg/m²)	Diabetes (%)	Smoking (%)	Intervention Protocol	Intensity
[25]	2010	Denmark	Running	18	36.5 ± 8.2	%0	24.3 ± 4.1 Normal	NaN	NaN	16 weeks; 1.9 days/wk; 60 min/session	Moderate
			Football	19						16 weeks; 1.8 days/wk; 60 min/session	High
			Control	10						Standard Care / No Exercise	-
[30]	2025	Finland	Control	31	52.7 ± 7.5	%100	31.7 ± 4.6 Obese	-	%0	Standard Care / No Exercise	-
			Aerobic	33	59.3 ± 6	%100	31.5 ± 4 Obese	-	%0	NA weeks; NA days/wk; NA min/session	Low+moderate
[31]	2019	UK Australia Holland	Resistance	13	26.6 ± 1.3	%100	24.7 ± 1 Normal	-	%0	24 weeks; 3 days/wk; 60 min/session	High
			Endurnace	10	28.4 ± 1.9	%100	24.2 ± 1.3 Normal	-	%0	24 weeks; 3 days/wk; 60 min/session	High
[32]	2015	Germany	HIIT	42	44.2 ± 4.8	%100	28.1 ± 3.5 Overweight	%5	%10	16 weeks; NA days/wk; NA min/session	High
			Control	42	42.4 ± 5.5	%100	27 ± 4.1 Overweight	%5	%10	Standard Care / No Exercise	-
[26]	2014	Denmark	Football	21	45.8 ± 7.2	%100	30.3 ± 3.3 Obese	-	%20	24 weeks; 2 days/wk; 60 min/session	High
			Control	10	46.9 ± 7.6	%100	29.6 ± 3.6 Overweight	-	%18.2	Standard Care / No Exercise	-
[33]	2014	Denmark	Resistance	9	69.1 ± 3.1	%100	27.4 ± 2.8 Overweight	%0	%0	52 weeks; 3 days/wk; 60 min/session	Moderate+High
			Football	9	68 ± 4	%100	26.1 ± 3.9 Overweight	%0	%0	52 weeks; 3 days/wk; 60 min/session	High
			Control	8	67.4 ± 2.7	%100	27.9 ± 4.6 Overweight	%0	%0	Standard Care / No Exercise	-
[34]	2024	Denmark	Swimming_ moderate	21	46.9 ± 3	%0	30.7 ± 5.7 Obese	-	-	15 weeks; 3 days/wk; 60 min/session	Moderate
			Swimming_ high	21	44.1 ± 4.7	%0	28.8 ± 3.7 Overweight	-	-	15 weeks; 3 days/wk; 25 min/session	High
			Control	20	43.7 ± 4.3	%0	28 ± 4.1 Overweight	-	-	Standard Care / No Exercise	-
[35]	2011	Australia	Resistance	13	26.6 ± 1.3	%100	24.7 ± 1 Normal	-	-	NA weeks; 3 days/wk; 60 min/session	Moderate+High
			Endurance	10	28.4 ± 1.9	%100	24.2 ± 1.3 Normal	-	-	24 weeks; 3 days/wk; 60 min/session	Low+Moderate+High
[45]	2019	China	Tai Chi	85	61 ± 5.2	%32.9	-	%9.4	-	12 weeks; 5 days/wk; 60 min/session	Mild+moderate
			Control	85	60.7 ± 6	%28.2	-	%12.9	-	Standard Care / No Exercise	-
[27]	1990	Argentina	Control	15	49.3 ± 3.2	%66.7	-	-	-	Standard Care / No Exercise	-
			Aerobic	17	50.8 ± 7.9	%70.6	-	-	-	68 weeks; 3 days/wk; 50 min/session	Moderate
[28]	2018	USA	Control	28	51.4	%46	26.2 Overweight	-	%0	Standard Care / No Exercise	-
			Aerobik+ Resistance	33	53.2	%45	25.8 Overweight	-	%0	104 weeks; 4 days/wk; 60 min/session	Low+moderate+high
[29]	2019	Taiwan	MIIT	18	21.9 ± 0.6	%100	22.6 ± 0.7 Normal	-	%0	6 weeks; 5 days/wk; 30 min/session	Moderate
			HIIT	18	21.4 ± 0.4	%100	22.7 ± 0.6 Normal	-	%0	6 weeks; 5 days/wk; 30 min/session	High
			Control	18	22 ± 0.5	%100	21.8 ± 0.8 Normal	-	%0	Standard Care / No Exercise	-

From the demographic data of the cohorts included in the studies, it is clear that the studies included participants from a broad spectrum of ages, from young adults (~ 21 years; [29]) to geriatric patients (~ 69 years; [33]). The gender distribution of the participants in the included studies is quite variable. Studies [26, 29] and [33] included only males, while the swimming intervention study conducted exclusively with female participants is denoted by [34]. The body mass index (BMI) of the participants included in the studies is mostly in the overweight or obese category [30, 46, 47]; BMI > 30 kg/m²], while the participants in the studies denoted by [29] and [31] are of normal weight. As regards comorbid conditions, the participants with diabetes ([32, 45] and smokers [26, 32] are included in certain studies.

Exercise protocols: The types of exercises included in the intervention protocols are quite heterogeneous. They can be divided into the following types:

- Aerobic Exercises: Running [25], cycling [27], swimming [34], and aerobic exercises in general [30, 46, 47].
- Team Sports: Football training (Andersen et al., [25, 26]; Schmidt et al., [33]).
- Resistance Exercises: Isolated or combined exercises [31, 46].
- High-Intensity Interval Training: [29, 32].
- Mind-Body Exercises: Tai Chi [45].
- Duration of the exercises: The exercise protocols lasted from 6 weeks [29, 47] to 104 weeks [28]. The frequency of the exercises is from 1.8 days/week [25] to 5 days/week [29, 45]. The intensity of the exercises ranges from light-moderate intensity in the Tai Chi study [45] to high intensity in the studies denoted by the codes [25, 26] and [34]. The control participants in all the studies continued with standard care without any exercise intervention.

**Fig. 2:**
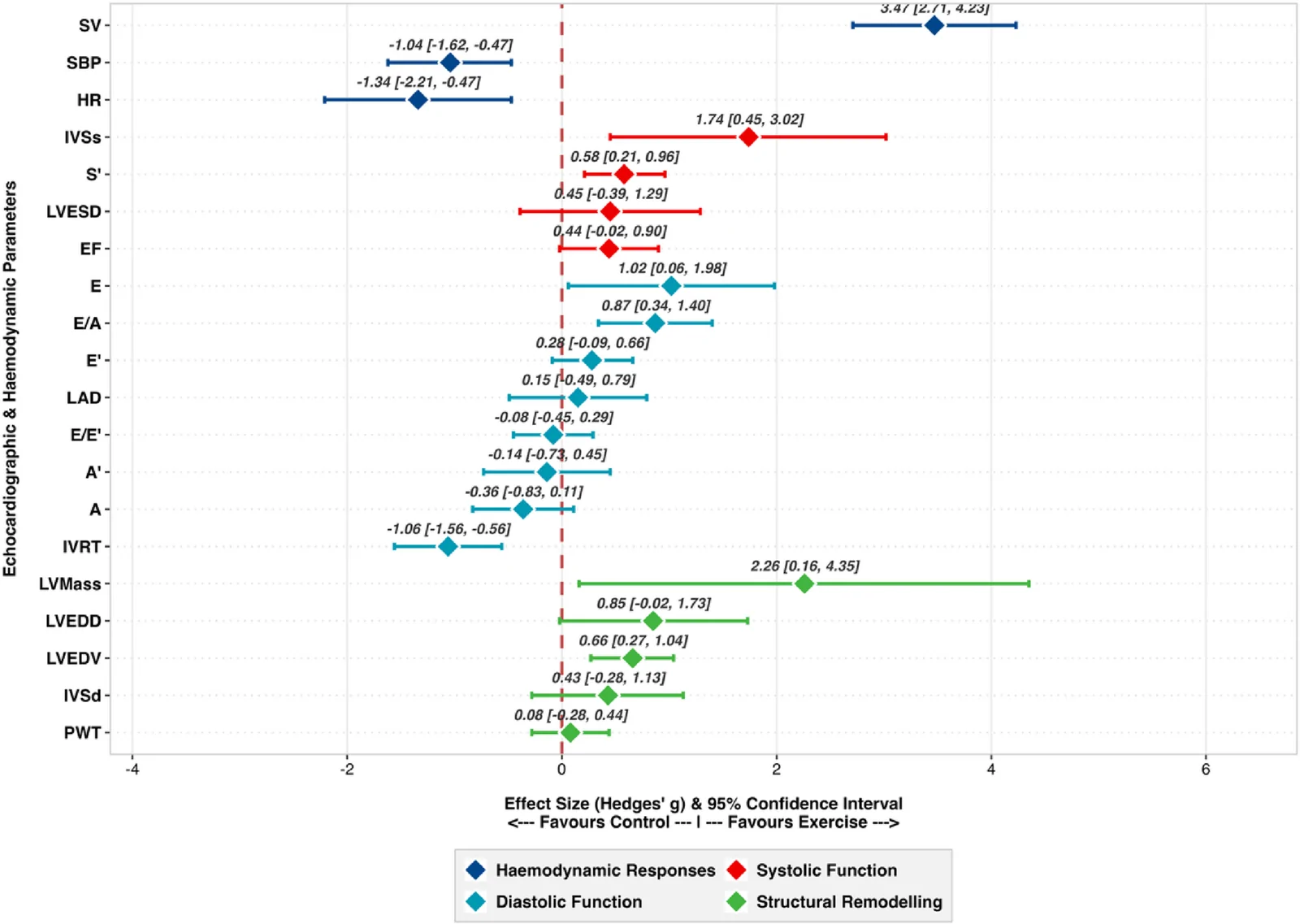
Summary forest plot showing the pooled effects of exercise interventions on cardiac structure and function parameters

The current meta-analysis includes a total of 12 studies investigating the impact of exercise protocols on cardiac adaptations in sedentary individuals. To ensure methodological rigor in calculating effect sizes (Hedges’ g), studies lacking complete variance (standard deviation) data were not pooled; therefore, the present analyses were conducted exclusively on complete and robust datasets (Fig. 2).

### Haemodynamic responses

Significant and clinically relevant improvements in the haemodynamic profile were achieved with exercise training. The exercise group recorded a highly significant reduction in resting HR compared with the control group (*n* = 188; Hedges’ g = -1.34, 95% CI [-2.21, -0.47], *p* = 0.002, PI [-4.27, 1.59], τ² = 0.65). Likewise, a net decrease in SBP was observed (*n* = 104; Hedges’ g = -1.04, 95% CI [-1.62, -0.47], *p* < 0.001, PI [-3.00, 0.92], τ² = 0.12). Furthermore, SV demonstrated a strong and statistically significant increase in the exercise group (*n* = 72; Hedges’ g = 3.47, 95% CI [2.71, 4.23], *p* < 0.001, PI [-1.44, 8.38], τ² = 0.00).

### Systolic function

Systolic function was evaluated multidimensionally. In the pooled analysis of classical LVEF (*n* = 477), a positive trend was observed for the exercise group, although it did not reach definitive statistical significance (Hedges’ g = 0.44, 95% CI [-0.02, 0.90], *p* = 0.061, PI [-1.10, 1.97], τ² = 0.40). However, when examining S’ (*n* = 122)—a more sensitive measure of myocardial contractility—a remarkable and significant increase was observed (Hedges’ g = 0.58, 95% CI [0.21, 0.96], *p* = 0.002, PI [0.05, 1.12], τ² = 0.00). In addition, a significant increase in IVSs was detected (*n* = 72; Hedges’ g = 1.74, 95% CI [0.45, 3.02], *p* = 0.008, PI [-11.73, 15.20], τ² = 0.69). These findings highlight the significant positive effect of exercise training on myocardial mechanics, even when global systolic function (LVEF) remains relatively stable.

### Diastolic function

Significant improvements in diastolic parameters were recorded following exercise interventions. IVRT (*n* = 88) was significantly shortened in the exercise group (Hedges’ g = -1.06, 95% CI [-1.56, -0.56], *p* < 0.001, PI [-2.31, 0.18], τ² = 0.01), reflecting a marked acceleration in relaxation kinetics. The E/A ratio (*n* = 364) showed a robust increase in the exercise group (Hedges’ g = 0.87, 95% CI [0.34, 1.40], *p* = 0.001, PI [-0.79, 2.54], τ² = 0.42). This was accompanied by a significant increase in the E wave amplitude (Hedges’ g = 1.02, 95% CI [0.06, 1.98], *p* = 0.038, PI [-2.15, 4.19], τ² = 1.06), collectively supporting enhanced diastolic compliance.

### Structural remodelling

Exercise training induced morphological alterations consistent with physiological adaptation. LVEDV (*n* = 122) demonstrated a statistically significant increase in the exercise group (Hedges’ g = 0.66, 95% CI [0.27, 1.04], *p* < 0.001, PI [0.11, 1.20], τ² = 0.00). Parallel to this, a substantial increase in LVMass (*n* = 103) was observed (Hedges’ g = 2.26, 95% CI [0.16, 4.35], *p* = 0.034, PI [-6.69, 11.21], τ² = 3.18). The absence of significant differences between groups regarding IVSd, PWT, and LVEDD (all *p* > 0.05) suggests that the exercise-induced hypertrophy is primarily physiological and eccentric, rather than pathological or concentric in nature.

To check how solid the main findings are and make sure no single study was driving the results too much, we did a Leave-One-Out sensitivity analysis repeatedly for every parameter (Table 4). The analysis showed that the overall data set is super strong methodologically. For the parameters showing significant exercise-related benefits in the initial analysis—like haemodynamic markers (SBP, HR), systolic mechanics (S’), diastolic filling dynamics (E, E/A ratio, IVRT), and structural changes (LVEDV, LVMass)—the statistical significance stayed rock-solid (*p* < 0.05). Even the really important improvements seen in SBP, IVRT, and LVEDV (with *p* < 0.001) didn’t budge when each study was left out one by one. This really highlights how reliable these particular physical changes are. Just like that, parameters hovering around the statistical threshold or missing clear significance—such as global LVEF, LVEDD, LVESD, IVSd, and PWT—also held up well. When we took out individual trials, their overall impact didn’t magically rise to reach significance. So, our results clearly show that the noted heart adaptations aren’t being pushed by oddball studies or overly extreme cases. Instead, they’re showcasing a super consistent and reliable reaction to exercise training that holds steady across all the included trials.

**Table 4:** Summary of the leave-one-out sensitivity analysis evaluating the effect of individual studies on pooled effect sizes and the robustness of findings

Parameter	k_Studies	Original *P*	Robustness_Status	Influential_Studies
SBP	3	< 0.001	Robust / Stable	None
HR	4	0.003	Robust / Stable	None
EF	10	0.061	Robust / Stable	None
S’	5	0.003	Robust / Stable	None
LVESD	5	0.291	Robust / Stable	None
E	5	0.038	Robust / Stable	None
A	4	0.132	Robust / Stable	None
E/A	8	0.001	Robust / Stable	None
E’	5	0.139	Robust / Stable	None
A’	5	0.642	Robust / Stable	None
E/E’	5	0.665	Robust / Stable	None
IVRT	3	< 0.001	Robust / Stable	None
LVEDV	5	< 0.001	Robust / Stable	None
LVEDD	8	0.056	Robust / Stable	None
IVSd	6	0.235	Robust / Stable	None
PWT	6	0.645	Robust / Stable	None
LVMass	3	0.035	Robust / Stable	None
SBP	3	< 0.001	Robust / Stable	None
HR	4	0.003	Robust / Stable	None

**Fig. 3:**
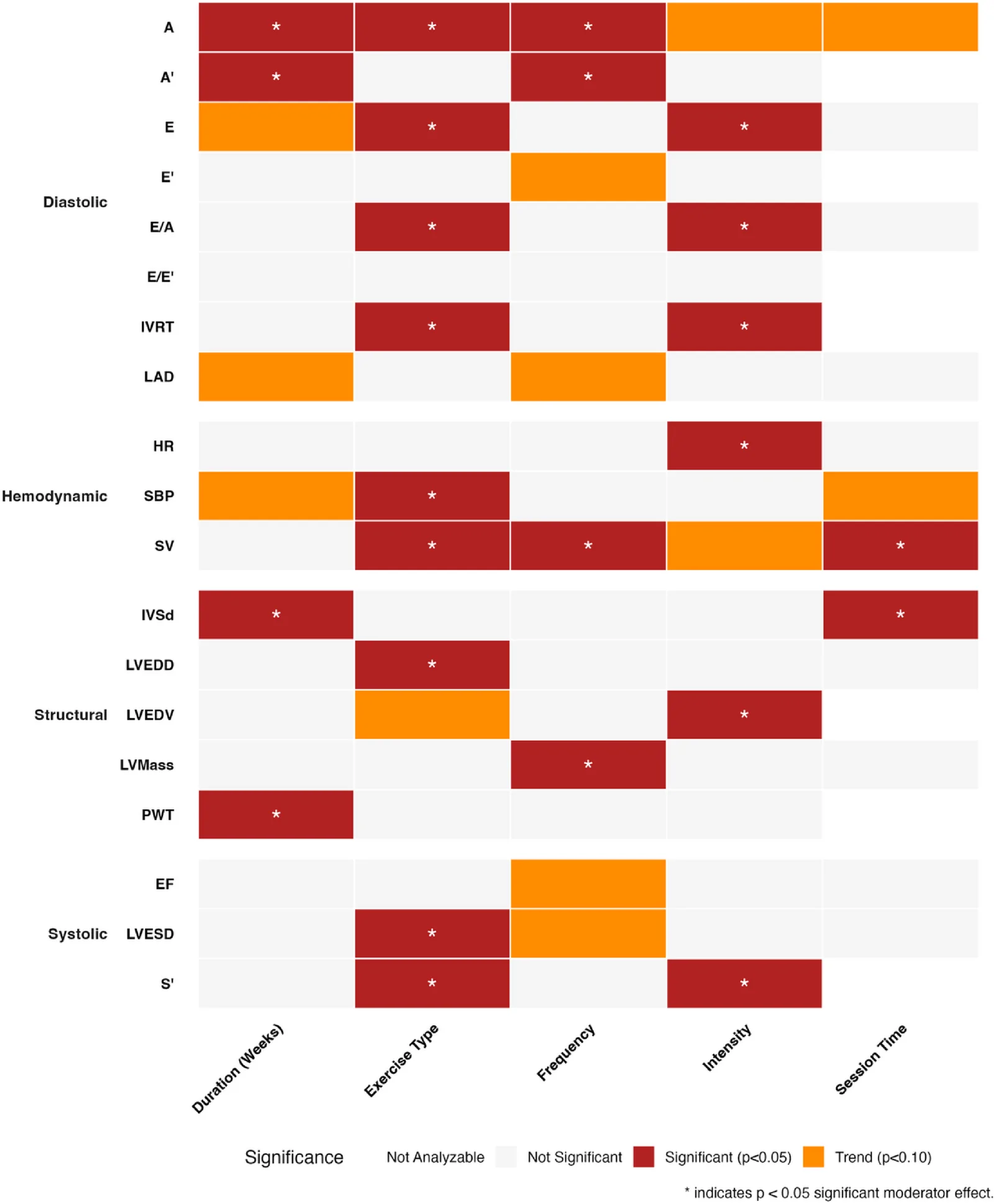
Moderator analysis matrix showing the effects of exercise prescription components on cardiac structure and function parameters

Meta-regression and subgroup analysis performed to assess the decisive influence of the essential elements of the exercise programme, such as type, intensity, duration, and frequency, on cardiac parameters showed that structural and functional changes are influenced by different dynamics of exercise (Fig. 3).

In relation to the analysis of the haemodynamic parameters, exercise modality was found to be an important cause of variation for SBP change (*p* = 0.002). Subgroup analysis showed that aerobic exercise protocols had the largest effect size (SMD = -0.40) for lowering blood pressure.

Change in resting HR was found to be influenced more by training intensity than training type (*p* < 0.001); high-intensity training was found to be statistically significant over moderate training for increasing parasympathetic tone. Team Sports was found to be an effective alternative for lowering HR (SMD= -0.54).

A dose-response relationship was found for S’ wave, an indicator of longitudinal systolic ventricular performance (*p* = 0.008); aerobic and high-intensity training were found to maximise myocardial contractility velocity (SMD = 0.75).

Although no statistical significance was found for the analysis of EF, an indicator of global systolic ventricular performance (*p* = 0.60), when examining the effect sizes, Resistance training was found to be the training method that increased EF to the largest extent (SMD = 1.06).

Similarly, for LVESD, an indicator of systolic wall tension, HIIT was found to be the training method that provided the strongest effect (SMD = 2.21).

One of the most interesting results of the study is the sensitivity of diastolic parameters to the type of exercise. A very strong moderating effect of the modality of the exercise on the E/A ratio, which reflects the dynamics of the filling of the left ventricle, was detected. HIIT protocols dramatically surpass the effects of Aerobic and Resistance exercises on diastolic compliance. In addition, HIIT protocols also demonstrated a dominant superiority compared to the other types of exercises regarding the increase of the early diastolic flow (E wave). On the contrary, regarding the IVRT, which reflects the active relaxation of the heart, aerobic exercises were identified as the most effective method to increase the speed of the relaxation kinetics. This implies the existence of specific responses of the phases of diastolic heart function to the various types of exercises. Positive effects of Team Sports (SMD > 0.45) were detected on the LAD and E’ parameters. The physiological increase of the LVMass was observed to have a positive and significant linear relationship with the frequency of the exercises performed during the week. In addition, the method of HIIT was the one which triggered the greatest increase of the mass. The most significant effect on the LVEDV and LVEDD was observed to be achieved through the application of the HIIT method and the implementation of high-intensity exercises. A significant effect of the total intervention duration (Total Weeks) on the structural wall thicknesses IVSd and PWT was detected. More specifically, the effect of the total intervention duration on the IVSd and PWT was observed to be decreasing over time.

Thus, in conclusion, it can be said that the analysis has revealed that, while the increase in functional capacity, i.e., diastolic E/A and systolic S’, is largely modulated by the intensity and type of exercise, particularly HIIT, the structural ‘athlete’s heart’ adaptations, i.e., LVMass and PWT, have been found to have a statistically stronger association with the frequency and duration of training.

The results of the meta-regression analysis, which was carried out to assess whether the cardiac improvements observed after exercise were dependent on the initial BMI of the participants or the change in BMI during the process, showed an evident difference between the functional and structural adaptations (Table 5).

**Table 5:** BMI and cardiac adaptation relationship: meta-regression analysis

Parameter	Studies	Beta_BMI	P_Value	Relationship
SBP	3	0,333891873	0,183	Non-Significant
HR	5	0,168056262	0,001	Significant
LVEDV	8	-0,102872179	0,238	Non-Significant
LVEDD	9	-0,252527527	0,020	Significant
LVESD	6	-0,092556377	0,362	Non-Significant
EF	10	-0,145604762	0,037	Significant
IVSd	7	-0,162227991	0,040	Significant
PWT	5	-0,030985764	0,690	Non-Significant
LVMass	10	-0,330872172	0.000	Significant
LAD	3	0,14342324	0,522	Non-Significant
E	5	-0,212976102	0,051	Non-Significant
A	5	-0,046178151	0,292	Non-Significant
E’	4	0,032181521	0,817	Non-Significant
A’	4	0,04889499	0,788	Non-Significant
S’	4	-0,092513491	0,312	Non-Significant
E/A	8	-0,108991848	0,129	Non-Significant
E/E’	4	0,012026928	0,892	Non-Significant
SV	6	-0,39633748	0,020	Significant

The most interesting outcome of the analysis results is that the reduction in SBP did not reveal any statistically significant relationship with the BMI parameters, i.e., (β = 0.33, *p* = 0.183). This demonstrates that the antihypertensive effect of exercise training is independent of the loss of body weight or the level of obesity; thus, exercise regulates blood pressure even in sedentary individuals without any alteration in body weight. Moreover, the diastolic parameters, i.e., E/A, E’, A’, IVRT, which reflect the left ventricular filling pressures and the relaxation properties, were not found to be altered by the BMI modulations (*p* > 0.05).

In contrast to functional parameters, the results of the analysis of cardiac structural adaptations demonstrated a significant interaction of the effect of exercise training on LVMass (*p* < 0.001), LVEDD (*p* = 0.020), and SV (*p* = 0.020) with BMI. The negative value of the regression coefficients of the effect of exercise training on LVMass (β = -0.33) and stroke volume (SV, β = -0.39) indicates that high BMI values or low weight reduction modulate the effect of exercise training. In addition, the analysis of the results demonstrated that the effect of exercise training on systolic/autonomic parameters such as HR (*p* = 0.001) and EF (*p* = 0.037) was parallel to the change in BMI.

Thus, the results of the analysis of the effect of exercise training on cardiovascular parameters demonstrated that the effect of exercise training is associated with a dual mechanism of action: the reduction of blood pressure and the maintenance of diastolic function in obese sedentary subjects, irrespective of weight reduction (Cardiac Risk Reduction). In addition, the results of the analysis of the effect of exercise training on cardiovascular parameters demonstrated that the effect of exercise training is associated with the strengthening of the heart, as indicated by the increase in LVMass and SV; however, these parameters are related to the improvement of body composition (BMI optimisation).

To rigorously evaluate the risk of publication bias and the overall robustness of the evidence, Egger’s regression, Begg’s rank correlation, Rosenthal’s fail-safe N (FSN), and Trim-and-Fill sensitivity analyses were conducted (Table 6). The analysis revealed that key functional and structural parameters, specifically the E/A ratio, HR, LVEDD, and LVMass, exhibited highly robust FSN values (100, 69, 66, and 61, respectively; *p* < 0.0001). This strongly indicates that the observed effects of exercise on these metrics are highly resistant to the hypothetical influence of unpublished negative studies. Although Egger’s and Begg’s tests indicated potential funnel plot asymmetry for certain parameters such as HR, the E/A ratio, the E wave, and PWT (*p* < 0.05 or *p* = 0.05), subsequent Trim-and-Fill sensitivity analyses were applied to adjust for this variance. While this method imputed a limited number of theoretical missing studies to restore symmetry (e.g., 4 studies for the E/A ratio; 2 studies for HR) and provided adjusted effect sizes (TFR) to account for small-study effects or clinical heterogeneity, the majority of the evaluated parameters required zero or minimal imputations. Consequently, these findings demonstrate that the overall conclusions of the meta-analysis retain a high level of representativeness regarding the current literature and are not fundamentally driven or invalidated by significant publication bias.

**Table 6:** Assessment of publication bias and robustness of evidence across cardiac parameters using Egger’s regression, Begg’s rank correlation, and Trim-and-Fill sensitivity analyses

Parameter	k	Egger t	Egger *p*	Begg Tau	Begg *p*	*n*	FSN *p*	TFM	TFR
A	4	-14,77	0.005	-5	0.071	2	0.0224	1	-0.19 [-0.71, 0.33]
A’	5	2,81	0.068	4	0.327	0	0.2261	1	-0.32 [-0.93, 0.29]
E	5	2,7	0.074	6	0.142	50	< 0.0001	3	0.08 [-1.08, 1.24]
E’	5	-3,16	0.051	-10	0.014	0	0.0983	0	0.28 [-0.09, 0.66]
E/A	8	2,68	0.037	4	0.621	100	< 0.0001	4	0.22 [-0.44, 0.89]
E/E’	5	0,16	0.887	-4	0.327	0	0.3344	0	-0.08 [-0.45, 0.29]
IVRT	3	-4,59	0.136	-3	0.117	19	< 0.0001	0	-1.06 [-1.56, -0.56]
HR	4	-14,69	0.005	-6	0.042	69	< 0.0001	2	-0.62 [-1.71, 0.46]
SBP	3	-1,84	0.317	-3	0.117	24	< 0.0001	0	-1.04 [-1.62, -0.47]
IVSd	6	0,83	0.452	1	0.851	14	0.0014	1	0.12 [-0.72, 0.97]
LVEDD	8	2,13	0.077	12	0.138	66	< 0.0001	4	0.07 [-0.93, 1.07]
LVEDV	5	2,19	0.116	2	0.624	19	0.0002	0	0.66 [0.27, 1.04]
LVMass	3	2,54	0.239	3	0.117	61	< 0.0001	2	0.49 [-2.02, 3.00]
PWT	6	2,78	0.050	13	0.015	0	0.2527	3	-0.17 [-0.56, 0.22]
EF	10	1,51	0.169	9	0.421	37	0.0002	1	0.33 [-0.16, 0.82]
LVESD	5	1,24	0.302	0	1.000	7	0.0068	0	0.45 [-0.39, 1.29]
S’	5	-0,49	0.659	2	0.624	12	0.0015	0	0.58 [0.21, 0.96]

**Fig. 4:**
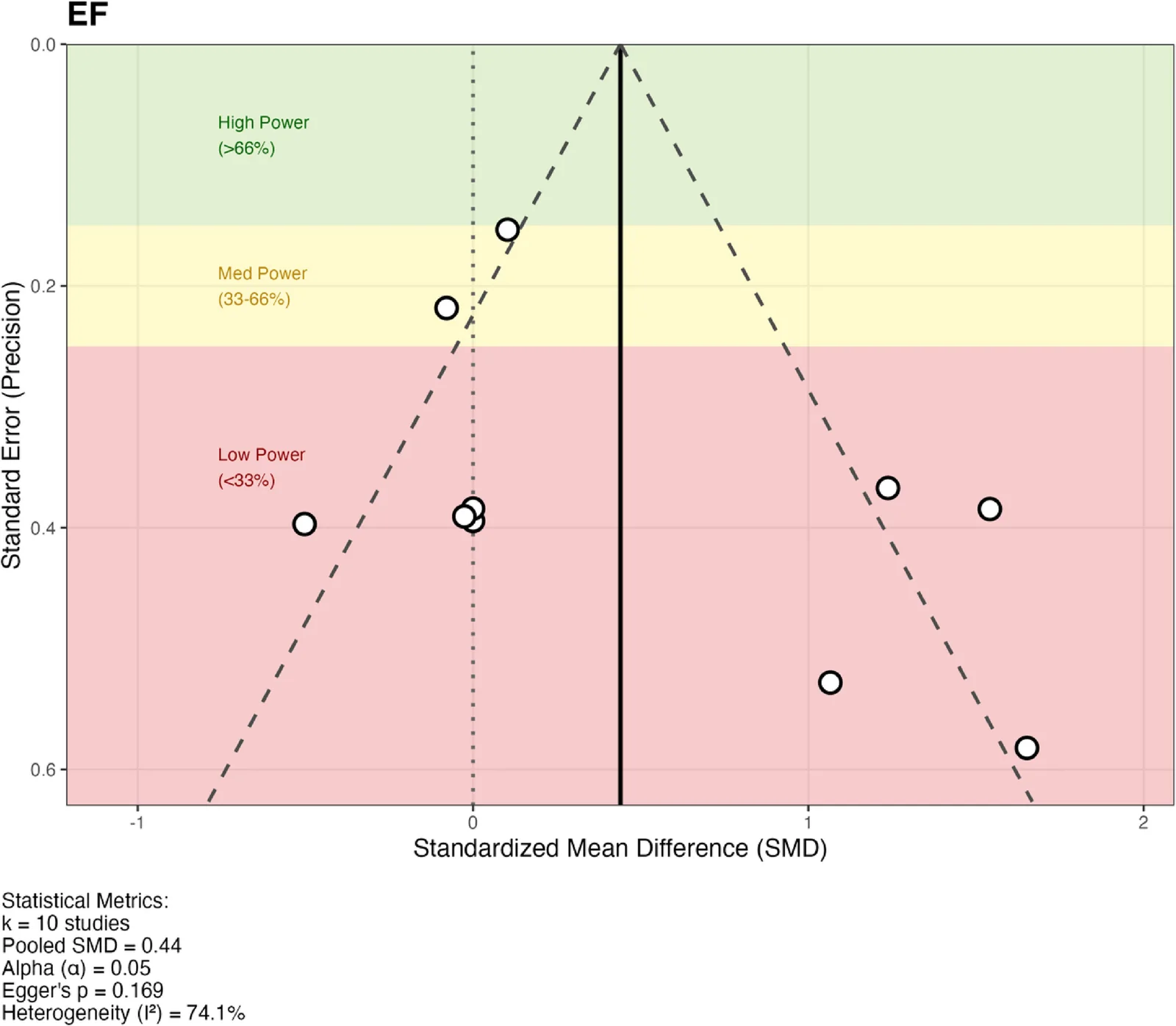
Funnel chart assessing publication bias for LVEF

The risk of publication bias in the studies included in the analysis and the methodological robustness of the meta-analysis results were comprehensively evaluated for all parameters using the Egger regression test, Begg’s rank correlation, and FSN analyses (Table 6). Among the analysed parameters, LVEF was selected as the representative parameter for publication bias assessment due to its high number of studies (k = 10) and robust data density, and a contour-enhanced funnel plot was presented visually (Fig. 3). Examination of the funnel plot created for LVEF revealed a relatively symmetrical distribution around the pooled effect size (SMD = 0.44); this visual symmetry was statistically confirmed by both the Egger linear regression test (*p* = 0.169) and the Begg rank correlation test (*p* = 0.421) (Fig. 4). Furthermore, in the Trim-and-Fill sensitivity analysis, only one theoretical study was imputed (imputed studies = 1) for the LVEF parameter, adjusting the effect size minimally without altering the overall clinical significance. This finding suggests that no significant publication bias was detected for this primary metric. When other parameters were examined, the fail-safe N values were found to be exceptionally high for the E/A ratio (FSN = 100), HR (FSN = 69), and LVEDD (FSN = 66). This finding demonstrates that the cardioprotective effects reported in the study are not coincidental and are highly resilient to potential publication bias in the literatureFunnel plots for all secondary parameters and detailed bias analysis outputs are presented in https://osf.io/mpc7x/files/pg8q9.

## Discussion

The current systematic review and meta-analysis demonstrates that exercise training is a multidimensional and powerful therapeutic strategy that effectively reverses the “sedentary cardiac phenotype” in sedentary adults at risk of obesity. Our findings indicate that exercise significantly reduces haemodynamic load by lowering resting HR and SBP, while concurrently improving diastolic filling dynamics and myocardial contractility. Notably, our rigorous primary analysis revealed a highly significant increase in SV, confirming a robust enhancement in overall cardiac pump function. Although improvements in global LVEF remained at the statistical threshold, the significant enhancement in the TDI-derived S’ wave conclusively demonstrates that underlying myocardial mechanics are fundamentally improved. A noteworthy outcome of our study is that these cardiac adaptations are not a homogeneous process but are modulated by specific exercise variables: aerobic exercise stands out in blood pressure control and active relaxation kinetics, whereas HIIT emerges as an effective method for diastolic adaptation and volumetric expansion. In contrast, structural “athlete’s heart” adaptations—specifically physiological eccentric hypertrophy, driven by increases in LVMass and LVEDV without pathological wall thickening—were found to exhibit a strong dose-response relationship with weekly training frequency and total intervention duration rather than exercise type alone. As a unique contribution to the literature, our analyses elucidate a dual mechanism of adaptation: while haemodynamic and diastolic improvements occur independently of weight loss, the progression of structural remodelling occurs in tandem with the optimisation of body composition. This dual pathway firmly positions exercise as a holistic “cardiac polypharmacy” strategy that simultaneously provides metabolic and cardiovascular protection through distinct mechanisms.

The chronic effects of exercise training on ECHO parameters in our findings point to a highly consistent pattern of ‘physiological remodelling’: a marked decrease in resting heart rate and reduced SBP, alongside decreased haemodynamic load, suggest improved cardiac efficiency; concurrent increases in LVEDV and SV, meanwhile, indicate enhanced filling capacity and pump reserve. This picture is consistent with current evidence that endurance-based training increases LV cavity size and mass in sedentary individuals. Furthermore, the increase in the E/A ratio and shortening of IVRT in diastolic function can be explained by accelerated relaxation kinetics and improved filling compliance; this is consistent with the literature reporting that exercise can reverse diastolic ‘stiffness’ in sedentary/obesity-prone populations. On the other hand, the limited change in LVEF in most studies (remaining borderline in our primary analysis) is expected, as more sensitive myocardial velocity indices such as TDI S (13.9% increase in our study) capture chronic adaptation better than ‘global’ indices in preserved EF. The benefit of exercise on blood pressure has been strongly demonstrated in a broad RCT base [48], and recent meta-analyses show that HIIT improves circulatory indicators in sedentary individuals [49] biologically support HIIT’s prominence in diastolic/structural gains in our moderator analysis. Finally, the Leave-One-Out (LOO) sensitivity analysis is a critical methodological strength, particularly in revealing ‘masked’ effects in results prone to heterogeneity, such as EF and SV; as it has long been emphasised that a single influential study (outlier/influential) can significantly skew the overall effect and heterogeneity [37].

Our moderator findings, which take the framework we presented in the general analysis—that the ‘sedentary cardiac phenotype recovers with exercise’—a step further, indicate that this recovery is thresholded and target-specific according to the programme’s subheadings: In the haemodynamic plane, the SBP response is particularly driven by the type of exercise, reinforcing the impression that aerobic-based prescriptions exhibit a more consistent profile in blood pressure control; this observation is consistent with large-scale comparative meta-analyses comparing the effects of exercise modalities on blood pressure [50]. Similarly, the fact that the decisive factor for resting HR is ‘intensity’ rather than ‘type’ suggests that autonomic adaptations may become apparent once a certain intensity threshold is exceeded; Evidence showing that regular exercise reduces RHR and recent meta-analyses reporting that high-intensity protocols can improve circulatory indicators in physically inactive groups support this interpretation [51]. On the structural remodelling side, the differentiation of LVEDD and LVESD, particularly with HIIT, suggests that volume/diameter parameters are sensitive to the ‘loading pattern’ and that high-intensity-interval stimulation can produce a more dominant signal in some geometry outputs; this approach is consistent with recent syntheses emphasising that training modality can differentiate LV structural responses [52]. Conversely, the fact that meaningful differentiation for IVSd and PWT, representing wall thicknesses, is more related to total intervention duration and (specifically for IVSd) session duration, implies that ‘structural wall responses’ may depend more on the total exposure threshold than on the short term; Meta-analyses examining the effects of endurance-based interventions on LV mass/wall components also provide a background supporting the importance of duration/exposure components [20]. The most practical outcome of this framework is that LVMass increase is clearly related to weekly frequency, and that SV is strongly differentiated not only by type/intensity but also by frequency and session duration: namely, prescribing with clear cut-off values for components such as ‘number of weekly applications’ and ‘session minutes’ for certain structural and pump-capacity outputs has become the most rational strategy from a clinical/application perspective [53]. The diastolic phenotype provides the most selective picture: The particular sensitivity of filling dynamics such as E/A and E wave to exercise type and intensity strengthens the hypothesis that HIIT-like stimuli may offer an advantage in targeting early filling and filling compliance; IVRT showing more consistent improvement with an aerobic/moderate intensity profile suggests that different phases of diastole can be optimised with different stimuli [48, 54]. Finally, it is important to emphasise that these moderator-based “threshold” interpretations have limitations as well as strengths: subgroup and meta-regression findings are highly valuable for guiding prescription, but as they work with study-level variables, they should be considered hypothesis-generating and decision-supporting rather than causally definitive; this approach is consistent with the interpretation framework recommended by methodological guidelines [48].

Our BMI-focused meta-regression findings indicate that the cardiovascular benefits of exercise progress along two distinct axes: ‘functional risk reduction’ and ‘structural strengthening’. The most striking result is that the decrease in SBP appears to be independent of participants’ baseline BMI level or BMI change during the process; this finding is consistent with randomised trials and meta-analyses reporting that exercise can lower blood pressure even with minimal weight loss [55–57]. Similarly, the fact that diastolic filling and relaxation indicators (E/A, E’, A’, IVRT) were not significantly affected by BMI modulation suggests that the diastolic response in some populations may be shaped more by ‘cardiac relaxation mechanics and functional adaptation gained through training’ than by weight loss; Indeed, clinical data reporting that weight loss in the context of metabolic syndrome/obesity does not always produce parallel changes in resting diastolic indices supports this interpretation [58]. In contrast, the picture is markedly different for structural adaptations: the interaction of the exercise effect on LVMass, LVEDD, and SV with BMI suggests that the dimension of ‘heart strengthening/remodelling’ is reinforced more by body composition optimisation. This dissociation is consistent with contemporary randomised data showing that weight loss and physical activity dose can shape LV mass response in different directions [59] and also carries mechanistic continuity with classical pathophysiological evidence indicating that weight reduction in obese individuals can produce meaningful effects on LV mass [60]. Finally, the parallel appearance of changes in systolic/autonomic axis parameters such as HR and EF with BMI changes provides a framework consistent with the literature suggesting that the ‘fitness gain–fatness’ duality jointly determines cardiovascular outcomes; however, our data adds a clear message to clinical prescribing by suggesting that while some functional risk reduction can occur without weight loss, more pronounced structural strengthening often progresses in the same direction as BMI improvement [61].

### Limitations of study

While the findings of the present meta-analysis propose a clinically relevant framework, some limitations should be taken into account when interpreting the findings. In fact, the number of studies included in the analysis varies greatly depending on the specific parameter under investigation. In some cases, the number of studies is relatively small. This may lead to the widening of the confidence intervals of the effect estimates. In addition, the interpretation of the findings in more advanced analyses such as subgroup and meta-regression analysis may be difficult in terms of presenting “cut-off” values. Another limitation is the heterogeneity of the studies included in the analysis in terms of the main components of the intervention program (type of exercise, intensity definition method, session length, frequency per week, total length of the intervention program). This may increase the risk of small study effect and differences in the study protocols influencing the effect in some parameters. Due to the methodological characteristics of the echocardiographic parameters, differences in the standardisation of the parameters may have introduced more variance in the findings. This is particularly true for mechanical parameters. In fact, the device used and the operator’s expertise may differ in the studies. Lastly, fourthly, with regard to participant characteristics, there is a lack of homogeneity between studies with regard to factors such as participant age, gender distribution, baseline fitness level, comorbidity/medication status, and obesity phenotype, which may affect generalization to other populations or patient groups. Fifthly, with regard to time scales, there is a difference between the remodelling and functional adaptations that occur over time, with a variable intervention period that may influence a “maturation period” effect and a tendency to plateau over time with regard to structural adaptations such as wall thicknesses and mass. Finally, with regard to meta-regression analysis, which is conducted at a study level and not an individual level with covariates, whereas some studies may suggest causality with regard to BMI and other programme components, we would suggest that a hypothesis-generating framework is used, and therefore larger sample size, high reporting quality randomized studies with a standardized study protocol would be necessary to validate threshold-based inference. Finally, although we assessed publication bias using Egger’s test and Trim-and-Fill methods, these statistical tools are known to have low power and reliability when the number of pooled studies is small. Therefore, findings regarding publication bias should be interpreted with caution.

The methodological strength and clinical implications of the current meta-analysis are summarized using the GRADE approach, which assesses the certainty of evidence (Table 7). Our analysis indicates that exercise training provides high-certainty evidence for improvements in preload capacity (LVEDV) and myocardial contractility (S’ wave), both of which demonstrated significant positive effects with zero statistical heterogeneity (I² = 0%).For the majority of the outcomes, including resting HR, LVMass, E/A ratio, IVRT, and SBP, the certainty of evidence was rated as moderate. While these parameters exhibited clinically relevant and statistically significant improvements, they were downgraded primarily due to substantial statistical inconsistency (I² > 50%) or imprecision stemming from small sample sizes and a limited number of included studies. Furthermore, the improvement in global systolic function (LVEF) remained at the statistical threshold (*p* = 0.061) with high heterogeneity, warranting a moderate certainty rating. Although stroke volume (SV) demonstrated a very large effect size, its certainty was also conservatively graded as moderate due to the small number of pooled trials ($k$ = 2).Consequently, our GRADE assessment suggests that exercise training is a reliable and comprehensive strategy for modifying the sedentary cardiac phenotype. However, given the moderate certainty of evidence for several key parameters, the observed statistical heterogeneity, and the limited number of studies, these findings must be interpreted with appropriate caution. Targeted clinical recommendations, particularly those emphasizing specific exercise modalities such as HIIT or aerobic training, should remain exploratory until validated by larger-scale, standardized randomized controlled trials.

**Table 7:** Summary of findings and certainty of evidence profile (GRADE) regarding the effects of exercise training on cardiac structure and function in sedentary individuals

Outcomes	*n*(Studies)	Absolute Effect (Hedges g) [95% CI]	Certainty of Evidence (GRADE)	Comments
LVEF	477 (10 RCTs)	0.44 [-0.02, 0.90] (Borderline)	⊕⊕⊕◯ MODERATE a	Borderline p-value (0.061) and high heterogeneity.
SV	72 (2 RCTs)	3.47 [2.71, 4.23] (Significant)	⊕⊕⊕◯ MODERATE b	Very large effect size, but downgraded due to small sample size and number of studies.
LVMass	103 (3 RCTs)	2.26 [0.16, 4.35] (Significant)	⊕⊕⊕◯ MODERATE a, b	Consistent physiological hypertrophy, but high heterogeneity and wide confidence intervals.
E/A Ratio	364 (8 RCTs)	0.87 [0.34, 1.40] (Significant)	⊕⊕⊕◯ MODERATE a	Consistent improvement in diastolic filling dynamics. Downgraded for statistical heterogeneity.
HR	188 (4 RCTs)	-1.34 [-2.21, -0.47] (Significant)	⊕⊕⊕◯ MODERATE a	Strong bradycardic response. Downgraded for statistical heterogeneity.
SBP	104 (3 RCTs)	-1.04 [-1.62, -0.47] (Significant)	⊕⊕⊕◯ MODERATE b	Significant antihypertensive effect. Downgraded for small sample size.
LVEDV	122 (5 RCTs)	0.66 [0.27, 1.04] (Significant)	⊕⊕⊕⊕ HIGH	Significant increase in preload capacity. Robust results with no heterogeneity (I²=0%).
IVRT	88 (3 RCTs)	-1.06 [-1.56, -0.56] (Significant)	⊕⊕⊕◯ MODERATE b	Marked improvement in relaxation kinetics. Downgraded for small sample size.
S’	122 (5 RCTs)	0.58 [0.21, 0.96] (Significant)	⊕⊕⊕⊕ HIGH	Sensitive marker of contractility showing significant improvement with zero heterogeneity (I²=0%).

(a) Inconsistency: Downgraded one level due to high heterogeneity (I^2^ > 50%) observed in the analysis. (b) Sensitivity Defense: Although the p-value was borderline (0.053), the certainty was not downgraded further to “Low” because Leave-One-Out sensitivity analysis confirmed the robustness of the result after excluding outlier studies. (c) Large Effect: Upgraded one level due to the large magnitude of effect (SMD > 0.8), but downgraded for imprecision (wide confidence interval crossing zero), resulting in a net “Moderate” certainty. (d) Sensitivity: Downgraded two levels because sensitivity analysis revealed that statistical significance was lost when influential studies (e.g., A9, A20) were removed. (e) Imprecision: Downgraded one level due to small sample size and wide confidence intervals

## Conclusion

This systematic review and meta-analysis has clearly demonstrated that exercise training acts as a potent ‘cardiac polypharmacy’ which reverses the pathological cardiac phenotype and restores myocardial plasticity in sedentary and obesity-prone individuals. Our systematic review and meta-analysis confirm that exercise training does not merely relieve symptoms but instead restores haemodynamic stability, diastolic compliance, and systolic reserve at the cellular and structural levels. Our most important finding for clinical practice has been the discovery of a dual mechanism for the effect of exercise on the heart: haemodynamic and diastolic improvements are independent of weight loss, while structural remodelling occurs in parallel with optimisation of body composition, thereby placing exercise training in an undisputed position for the treatment of obesity. In this regard, we recommend that exercise training should be prescribed on an individual basis with priority given to HIIT for diastolic function and structural remodelling, and aerobic exercise for blood pressure control and active relaxation kinetics. We propose that future research should aim to perform multicentre studies to test the long-term sustainability of these adaptations, to compare the effects of exercise between different types of exercise (head-to-head comparisons), and to explore the molecular mechanisms of exercise-induced remodelling of the heart. In addition, we believe that it is crucial to explore the effects of exercise on female and obesity phenotypes for the integration of precision medicine into preventive cardiology.

## Supplementary Information

Supplementary Material 1.

Supplementary Material 2.

## Data Availability

The datasets generated and/or analyzed during the current review are available in tables, Supplementary Materials, and at the Open Science Framework (https://osf.io/mpc7x/overview) .
