# Supplementary material for: Exercise reverses the sedentary cardiac phenotype in obesity: a systematic review and meta-analysis of hemodynamic, structural, and functional adaptations
Source: BMC Sports Sci Med Rehabil. 2026 Jun 24;18:404. doi: 10.1186/s13102-026-01822-6 (PMC13556015; doi:10.1186/s13102-026-01822-6)
Supplement: Supplementary file 2 [file 13102_2026_1822_MOESM2_ESM.docx]

| Database | Coding Strategy / Exact Search String | Number of Hits (n) | Date of last access |
| --- | --- | --- | --- |
| PubMed (MEDLINE) | ("sedentary"[tiab] OR "sedentary lifestyle"[tiab] OR "physical inactivity"[tiab]) AND ("exercis*"[tiab] OR "aerobic training"[tiab] OR "resistance training"[tiab] OR "endurance training"[tiab] OR "HIIT"[tiab]) AND ("Echocardiography"[Mesh] OR "echocardiograph*"[tiab] OR "cardiac remodeling"[tiab] OR "left ventricular mass"[tiab]) AND ("cohort"[tiab] OR "longitudinal"[tiab] OR "prospective"[tiab]) | 200 | 04.12.2025 |
| Scopus | TITLE-ABS-KEY ( ( "sedentary" OR "sedentary lifestyle" OR "physical inactivity" ) AND ( "exercise*" OR "aerobic training" OR "resistance training" OR "HIIT" ) AND ( "echocardiograph*" OR "cardiac remodeling" OR "left ventricular mass" ) AND ( "cohort" OR "longitudinal" OR "prospective" ) ) | 210 | 25.11.2025 |
| Web of Science (Core Collection) | TS=(("sedentary" OR "sedentary lifestyle" OR "physical inactivity" OR "inactive" OR "untrained") AND ("exercise*" OR "exercise training" OR "endurance training" OR "aerobic training" OR "resistance training" OR "strength training" OR "high-intensity interval training" OR "HIIT") AND ("echocardiograph*" OR "cardiac ultrasound" OR "left ventricular mass" OR "cardiac remodeling" OR "cardiac remodelling" OR "cardiac morphology" OR "ventricular function") AND ("cohort" OR "longitudinal" OR "prospective" OR "follow-up") AND ("adult" OR "adults" OR "middle-aged")) | 50 | 26.11.2025 |
